# Identifying genes associated with brain volumetric differences through tissue specific transcriptomic inference from GWAS summary data

**DOI:** 10.1186/s12859-022-04947-w

**Published:** 2022-09-28

**Authors:** Hung Mai, Jingxuan Bao, Paul M. Thompson, Dokyoon Kim, Li Shen

**Affiliations:** 1 Perelman School of Medicine, University of Pennsylvania, B306 Richards Building, 3700 Hamilton Walk, Philadelphia, PA USA; 2 School of Engineering and Applied Science, University of Pennsylvania, Philadelphia, PA USA; 3 School of Arts and Sciences, University of Pennsylvania, Philadelphia, PA USA; 4 Imaging Genetics Center, Stevens Institute for Neuroimaging and Informatics, Keck School of Medicine, University of Southern California, Los Angeles, CA USA

**Keywords:** Genetic variation, Gene expression, Brain imaging, Imaging genomic association, Brain volume

## Abstract

**Background:**

Brain volume has been widely studied in the neuroimaging field, since it is an important and heritable trait associated with brain development, aging and various neurological and psychiatric disorders. Genome-wide association studies (GWAS) have successfully identified numerous associations between genetic variants such as single nucleotide polymorphisms and complex traits like brain volume. However, it is unclear how these genetic variations influence regional gene expression levels, which may subsequently lead to phenotypic changes. S-PrediXcan is a tissue-specific transcriptomic data analysis method that can be applied to bridge this gap. In this work, we perform an S-PrediXcan analysis on GWAS summary data from two large imaging genetics initiatives, the UK Biobank and Enhancing Neuroimaging Genetics through Meta Analysis, to identify tissue-specific transcriptomic effects on two closely related brain volume measures: total brain volume (TBV) and intracranial volume (ICV).

**Results:**

As a result of the analysis, we identified 10 genes that are highly associated with both TBV and ICV. Nine out of 10 genes were found to be associated with TBV in another study using a different gene-based association analysis. Moreover, most of our discovered genes were also found to be correlated with multiple cognitive and behavioral traits. Further analyses revealed the protein–protein interactions, associated molecular pathways and biological functions that offer insight into how these genes function and interact with others.

**Conclusions:**

These results confirm that S-PrediXcan can identify genes with tissue-specific transcriptomic effects on complex traits. The analysis also suggested novel genes whose expression levels are related to brain volumetric traits. This provides important insights into the genetic mechanisms of the human brain.

**Supplementary Information:**

The online version contains supplementary material available at 10.1186/s12859-022-04947-w.

## Background

Brain volume changes throughout life and varies considerably across different individuals [1]. Abnormal changes in brain volume are also associated with several neuropsychiatric and degenerative disorders [2]. The variation in human brain volume can be studied with MRI. Specifically, whole brain MRIs are obtained and can be segmented into different pre-defined regions of interest (ROIs) for ROI-based volumetric analysis [2]. Brain ROI volumes are also highly heritable. The heritability of some ROI volumes is over 80%, based on data from twin studies, and it varies for different ROIs [3–8]. For example, heritability can range from 60 to 85% for different ROIs in the basal ganglia, limbic and diencephalic regions [7]. Variation in human brain volume is also associated with common genetic variants, called single-nucleotide polymorphisms (SNPs). In aggregate, SNPs account for over 50% of the variation in brain volumetric traits [9–13]. Therefore, genome-wide association studies (GWAS) of brain imaging phenotypes have been conducted to localize specific SNPs associated with phenotypic variation in brain structural and functional traits [2, 10, 13–15].

GWAS, however, cannot provide complete information on molecular mechanisms (such as gene expression alterations) underlying the connections between SNPs and complex traits. This represents a big gap for therapeutic development, as treatments often aim to target disease processes at the transcriptional level. Moreover, most of the SNP heritability (over 90%) is explained by noncoding variants, mainly in regulatory regions [16]. This further highlights the importance of understanding the association of gene expression regulation and the resulting phenotypes. For that reason, PrediXcan, a gene-based association analysis, was developed [17], to integrate SNP-based gene expression prediction models with GWAS analysis. The prediction model imputes the gene expression level based on all the SNPs within the gene. By using these predicted gene expression measures, PrediXcan can be employed to identify genes whose expression levels are associated with a phenotype, overcoming the limitations of GWAS. By mapping a large number of SNPs (e.g., over a million) to a moderate number of genes (e.g., less than twenty thousand), the PrediXcan strategy can greatly reduce the burden for multiple comparison and thus potentially increase detection power. On the other hand, given the tissue-specific nature of gene expression, different prediction models can be constructed to link gene expression data with phenotype traits for different tissue types [17].

Another advantage of PrediXcan is that it can be integrated into meta-analysis studies that aggregate GWAS results from multiple cohorts [17]. These meta-analyses generate “GWAS summary data” that can identify associations not detectable with smaller sample sizes. Methods such as S-PrediXcan were developed to harness the power of large data samples while keeping the computational burden at a manageable level [18]. S-PrediXcan is similar to PrediXcan as both can identify genes whose expression measures are associated with phenotypic traits. S-PrediXcan differs from PrediXcan as it proposes an analytic strategy to allow the use of GWAS summary statistic data instead of individual level data [18].

**Fig. 1:**
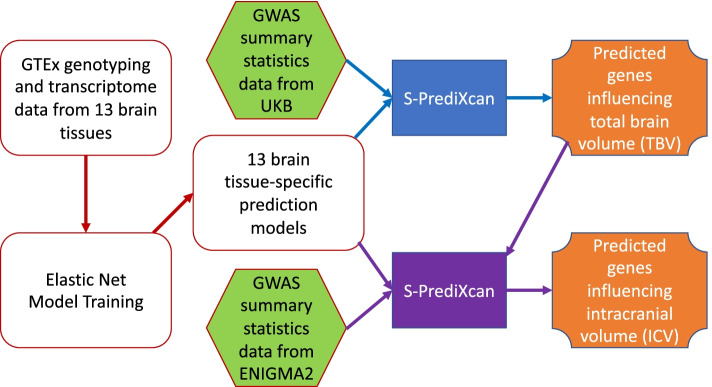
Schematic diagram describing the pipeline of this study which used S-PrediXcan to predict genes that are highly associated with total brain volume (TBV) and intracranial volume (ICV). S-PrediXcan integrates two inputs, one of them was trained PrediXcan elastic-net prediction models which derived from GTEx genotyping and transcriptome data of 13 brain tissues. The other inputs were GWAS summary statistics data of our interested traits: (1) TBV from UKB and (2) ICV from ENIGMA2. The first S-PrediXcan analysis on UKB data yielded predicted genes that are highly associated with TBV. The second S-PrediXcan analysis aimed to perform a targeted study on a similar trait (ICV) using the GWAS summary data from an independent cohort (ENIGMA2) to determine which TBV-associated genes are also significantly associated with ICV

In this study, we propose to use S-PrediXcan to integrate the GWAS summary statistics of an imaging trait with PrediXcan models linking SNPs to gene expression data in a specific brain tissue. The goal is to detect genes whose expression levels have mediating effects on the imaging trait. Specifically, we perform S-PrediXcan analysis (Fig. 1) using the brain imaging GWAS summary data for total brain volume (TBV) and intracranial volume (ICV) from two landmark studies: (1) UK Biobank (UKB) [2, 10, 19] and (2) Enhancing Neuroimaging Genetics through Meta Analysis (ENIGMA) [13, 14, 20]. These GWAS summary data are integrated with 13 brain tissue specific prediction models from the Genotype-Tissue Expression project (GTEx) [21] to identify genes whose expression levels are highly associated with both ICV and TBV. We aim to identify promising gene discoveries that can provide important information to help us better understand the molecular processes that shape the human brain.

## Results

### Tissue-specific transcriptome analysis identified 10 genes that are highly associated with both TBV and ICV

**Fig. 2:**
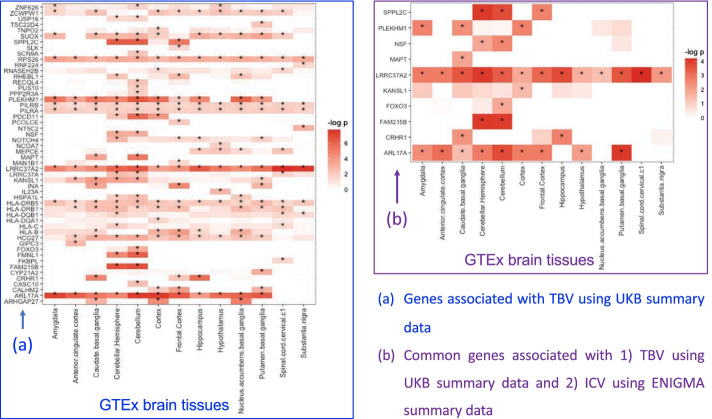
Genes that are highly associated with brain volumes based on S-PrediXcan analysis. **a** Genes that are highly associated with TBV using the UKB GWAS summary statistics. **b** Common genes that are associated both with TBV using the UKB GWAS summary statistics and with ICV using the ENIGMA summary statistics. Entries marked with * are significant tissue-specific gene-phenotype associations (FDR < 0.05), where 13 GTEx brain tissues are plotted on the *x* axis

In this study, we conducted tissue specific transcriptomic analysis by using S-PrediXcan to predict genes that were potentially correlated with brain volumetric measures. We first performed the analysis by using the GWAS summary data from the UKB cohort and identified 208 significant gene-TBV associations, which involved 52 genes and 13 brain tissues (Fig. 2a, Additional file 1: Table S1a). To determine whether these 52 genes would also be associated with ICV (a relevant brain volume measure), we ran S-PrediXcan again by using GWAS summary data from the independent ENIGMA2 cohort. We observed that 10 out of 52 genes associated with TBV were also associated with ICV (Fig. 2b, Additional file 1: Table S1b). The results indicate these 10 genes (SPPL2C, PLEKHM1, NSF, MAPT, LRRC37A2, KANSL1, FOXO3, FAM215B, CRHR1, ARL17A) may contribute to the molecular basis of the brain volumetric measures, and some are been associated with cognitive and mental health traits.

### Concordance with prior studies and functional mapping of genes highly associated with ICV and TBV

Among these 10 genes, 9 of them (except FAM215B) were significantly associated with TBV in the gene-based association analysis of the original UKB GWAS (Additional File 1: Table S2) [2]. While the original UKB analysis revealed the significant collective effect of SNPs within each of these genes, our analysis identified the mediating effects of the expression levels of these genes not only on TBV (in UKB) but also on ICV (in the independent ENIGMA2 cohort). In addition, our S-PrediXcan analysis also yielded valuable tissue specificity information, revealing varying mediating effects of these genes across different brain tissues (Fig. 2b). Our analysis also identified a new gene (FAM215B), not found in prior studies, that was highly associated with brain volume development.

**Fig. 3:**
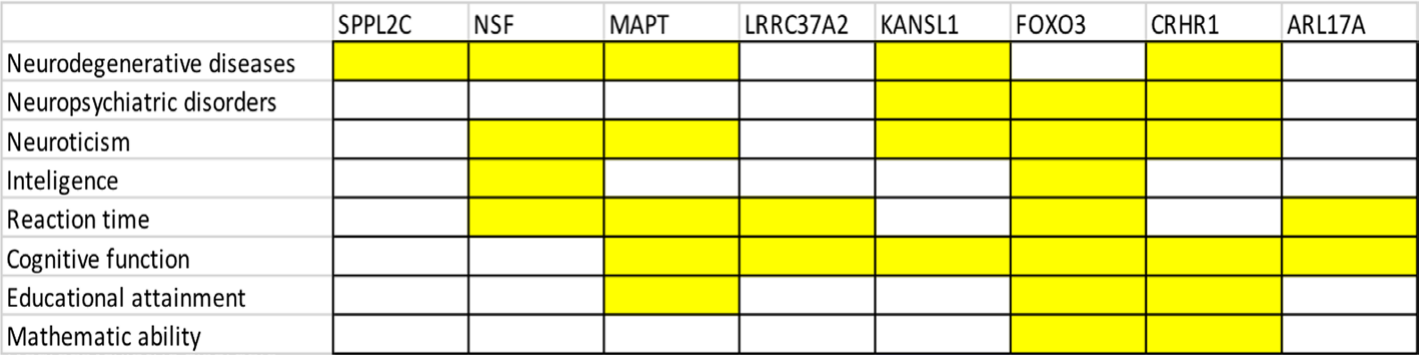
Eight of ten genes that are commonly associated with TBV and ICV have also been reported to relate with several cognitive functions and mental health disorders (highlighted blocks). This analysis was performed by manually searching for our reported genes in the NHGRI-EBI human GWAS catalog and recording their associations with traits related to cognitive function and mental health conditions

By comparing our results to prior GWAS findings (including those in the NHGRI-EBI GWAS catalog, https://www.ebi.ac.uk/gwas/), 8 out of 10 genes were found to be correlated with different cognitive and behavioral traits (Fig. 3). Those traits and their associated genes include: neurodegenerative diseases (SPPL2C, NSF, MAPT, KANSL1, CRHR1), neuropsychiatric disorders (KANSL1, FOXO3, CRHR1), neuroticism (NSF, MAPT, KANSL1, FOXO3, CRHR1), intellectual performance (NSF, FOXO3), reaction time (NSF, MAPT, LRRC37A2, FOXO3, ARL17A), cognitive function (MAPT, LRRC37A2, KANSL1, FOXO3, CRHR1, ARL17A), educational attainment (MAPT, FOXO3, CRHR1) and mathematical ability (FOXO3, CRHR1). This further supports our hypothesis in which our 10 reported genes are important in determining brain structures and functions.

### Molecular investigation of the network of genes associated with ICV and TBV

**Fig. 4:**
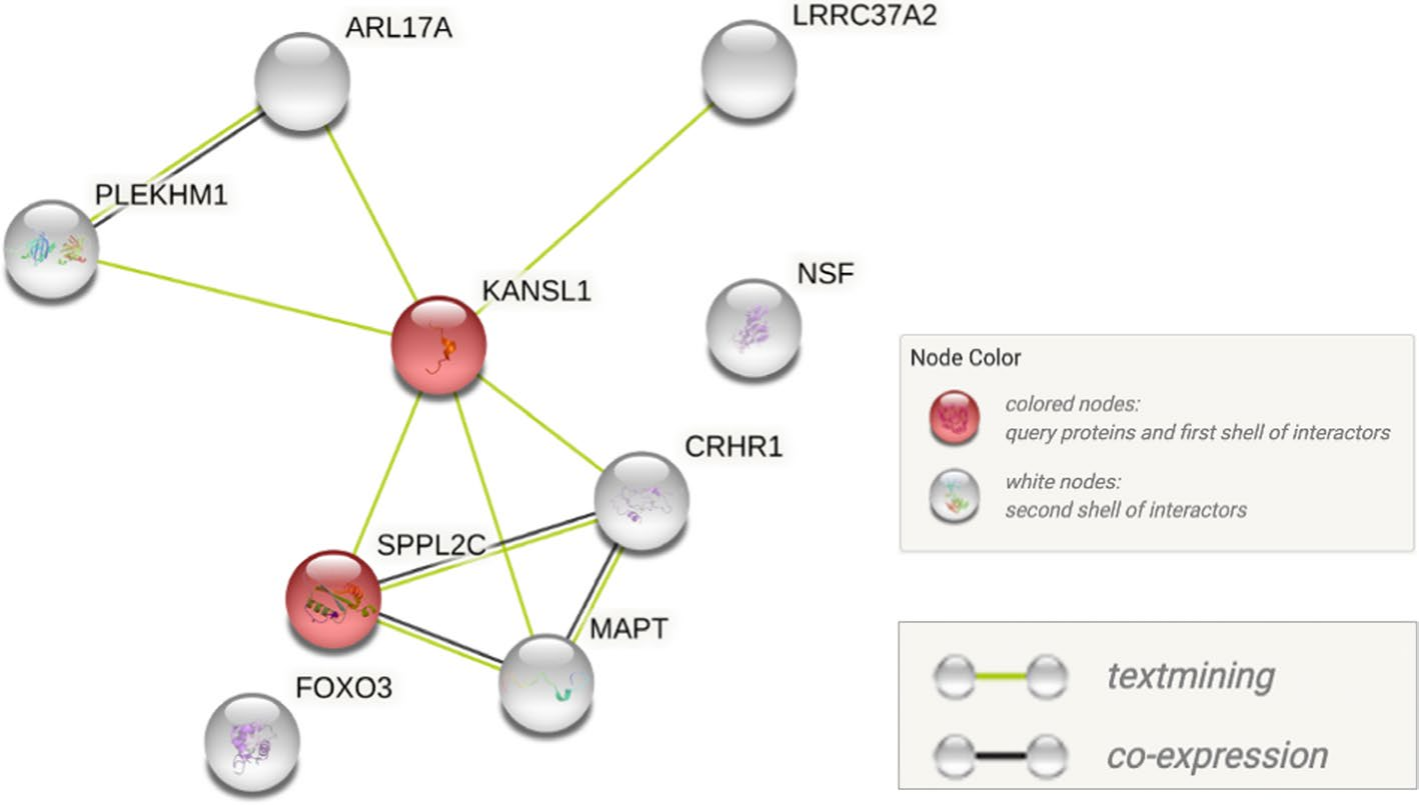
Protein interaction network created by the STRING software. As FAM215B does not exist in the STRING database, the network includes nine out of ten reported genes that are associated with both TBV and ICV. Each node represents each protein-coding gene. Nodes are connected by edges that represent known associations between proteins. Colored nodes indicate the first shell of interactors, and white nodes indicate the second shell of interactors. Edges with different colors represent different sources used to obtain the information on protein associations

The interaction network of proteins coded by our discovered set of genes was generated using STRING (https://string-db.org/). Among 10 genes, 9 of them (except FAM215B) were represented in the database. 7 out of 9 genes were found to interact with the others (Fig. 4). KANSL1 and FOXO3 were found to be the first shell of interactors. Two sources used to obtain the protein interaction were “text-mining” and “co-expression”. In STRING database, each protein–protein interaction has the corresponding interaction score which indicates the confidence of the predicted interaction. The scores range from 0 to 1 with 1 indicating the highest level of confidence, and 0.5 indicating that there’s a roughly 50% chance that the predicted interaction could be correct. All the protein–protein interactions in Fig. 4 range from 0.4 to 0.7, which indicates an intermediate level of confidence [22]. A knowledge of protein interaction provides valuable information for further understanding of how these genes work together to influence brain volume and affect brain-related traits.

**Fig. 5:**
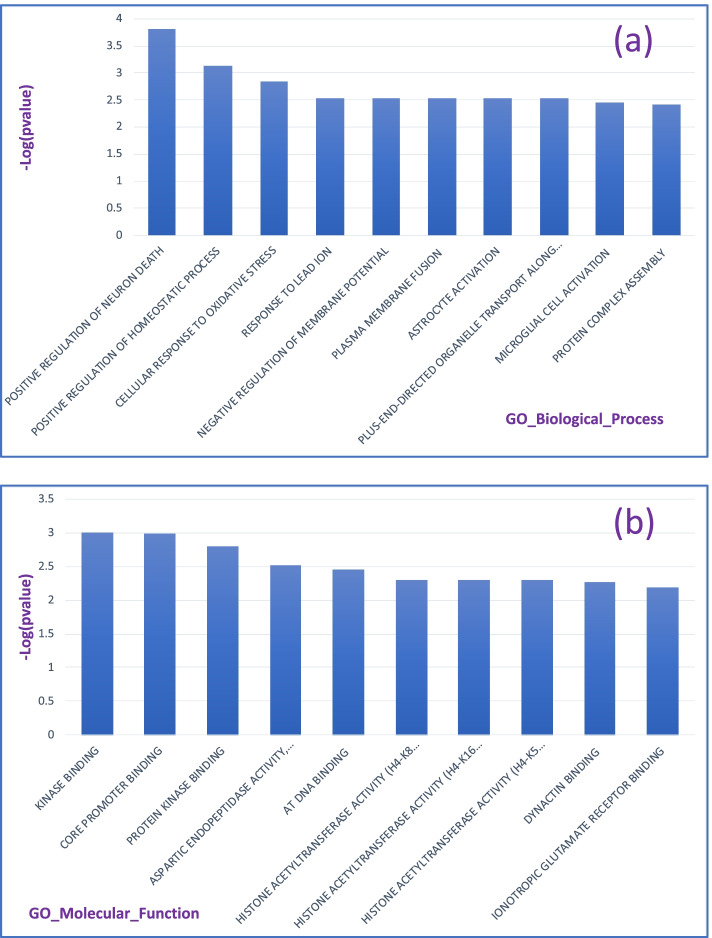
GO analysis of the 10 genes that are found to highly associated with TBV and ICV. **a** Top 10 GO biological processes that are enriched in our set of 10 discovered genes. **b** Top 10 molecular functions that are enriched by our 10 discovered genes. The *x* axis represents the name of each biological process or molecular function. The y axis represents the -log (*p* value), where *p* value comes from the enrichment analysis of our 10 reported genes by using https://maayanlab.cloud/Enrichr/

Gene Ontology (GO) enrichment analysis was also performed by using 2 different tracks: GO Molecular Function and GO Biological process (https://maayanlab.cloud/Enrichr/). These analyses suggested some biological processes that were associated with the 10 genes, including positive regulation of neuronal death, astrocyte activation, microglial cell activation, among others (Fig. 5a, Additional file 1: Table S3). These biological processes are all related to several central nervous system pathologies such as trauma, stroke, or neurodegenerative diseases. GO analysis also suggested some other biological pathways potentially correlated with the roles of these 10 genes, including cellular response to oxidative stress, negative regulation of membrane potential, positive regulation of homeostatic process, etc. The molecular functions associated with these genes were also determined including kinase binding, core promoter binding, and histone post-translational modifications (Fig. 5b, Additional file 1: Table S4). It should be noted that many of the enriched terms resulted from the overlapping of only one or two genes which might not be precise enough for the conclusion. However, we hope they could shed a light for the future studies on molecular mechanisms that are important to the process of brain development and diseases.

## Discussion

Changes in brain volumes are known to be correlated with various neurological and psychiatric problems such as cognitive/behavioral defects and neurodegenerative diseases [2], and thus are important topics for biomedical investigation. Brain volumes are traits that are highly heritable, and the genetic factors play key roles in brain volume changes and account for more than 50% of the variations in brain volume among individuals [9–13]. Many studies including GWAS have been performed and identified multiple SNPs correlated with human brain volumes and structures [2, 10, 13–15]. Most of the prior studies, however, were based on small sample sizes and thus might have limited statistical power to identify all the relevant SNPs, especially those with small effect sizes [10, 23–27]. Recently, there have been multiple landmark GWAS projects of large sample sizes (more than 15,000 participants) to identify SNPs that are associated with different brain volumetric measures [2, 13] In this study, we aimed to harness the power of these SNP-trait association data which was generated from large-scale studies to reveal genes highly associated with brain volume differences. We used tissue-specific transcriptomic analysis (SPrediXcan) [18] and identified 10 genes that are highly associated with ICV and TBV. Our study, on one hand, confirmed SPrediXcan as a powerful tool to identify genes that are associated with complex traits. On the other hand, we conducted post-hoc pathway and network analysis to provide insights into transcriptomic regulation and profile underlying brain volumetric phenotypes. Based on our results, further investigations can be performed to gain deeper understanding into the biological mechanisms of brain structure and function, and subsequently impact the study of neurological and psychiatric disorders.

Our study has faced a few challenges and limitations. First, our analysis depends on the GWAS summary data that were generated by other studies. Since SPrediXcan requires the inputs to have a strict format, we worked with the GWAS summary data meeting this requirement. This was somewhat challenging because not all the GWAS studies shared their summary data satisfying SPrediXcan’s requirement. On the other hand, the traits studied in the UKB and ENIGMA studies were related but not exactly the same, and thus we would not be able to perform a replication study. Instead, we focused on analyzing two closely related brain volume measures ICV and TBV [2, 13].

Second, we used the GTEx data which was from healthy samples. Thus, this study aimed to detect genes that play important roles in the normal development process of the brain. In order to study genes that are related to pathological changes in the brain, an interesting future direction could be to perform similar analyses on the disease-related biobank data such as those from the PsychENCODE consortium (https://psychencode.org/).

Third, in this work, we examined only the PrediXcan models trained by the elastic-net method. An interesting future direction would be to include also the MASHR-based PrediXcan models and perform a more comprehensive comparative study. For example, we can benchmark these models, tune relevant parameters, and seek for an improved PrediXcan model that identifies more genes.

Fourth, this work is a pure bioinformatics study, and thus no molecular experiments are performed to validate the findings. However, our goal is to identify promising genes for subsequent replication study in independent cohorts as well as form new hypothesis for molecular validation. For example, one future direction could be to conduct knockout experiments in mice with some of the genes we discovered, especially with the gene that has not been heavily studied before (e.g., FAM215B). By knocking out these genes, alone or in combination with the others, one can observe the changes in brain volumes in mice. Another direction could be to perform molecular experiments (e.g., RNA sequencing, Chromatin immunoprecipitation sequencing, mass spectrometry) on relevant brain tissues. These investigations will further evaluate and reveal the underlying molecular networks of how these genes function and how they contribute to the changes of brain volumes and structures.

## Conclusions

We performed tissue-specific transcriptomic association analyses using S-PrediXcan on the UKB and ENIGMA2 GWAS summary data. We identified 10 genes with varying mediating effects on both total brain volume (TBV) and intracranial volume (ICV) across thirteen GTEx brain tissues. We examined our results by comparing them to the findings of prior GWAS studies and found that 8 out of 10 genes were correlated with cognitive and behavioral deficits in humans. Moreover, 9 out of 10 genes were found to be associated with TBV in another study using a different gene-based association analysis [2]. In the current study, we also included further post-hoc analyses to reveal possible biological and cellular mechanisms as well as the interaction network of proteins coded by the discovered genes. These identified genes, coupled with their tissue specific findings, warrant further investigation in independent cohorts. Molecular validation is also needed, to better understand molecular mechanisms of the brain and brain disorders such as Alzheimer’s diseases, and ultimately to potentially aid in therapeutic strategy development.

## Methods

### Data and materials

We performed our analyses using the imaging GWAS summary data from two landmark studies (UKB [2] and ENIGMA [13]), to leverage the statistical power provided by their large sample sizes. Both studies have yielded many imaging genetic associations that could not be detected with smaller sample sizes.

In the first study, we analyzed the GWAS summary data generated by Zhao et al. [2], where they aimed to identify SNPs associated with multiple brain volumetric phenotypes from the UKB cohort. Although previous similar studies were done with the same purposes, they all analyzed data sets with small sample size, and might have missed SNP-phenotype associations with small effect sizes [10, 23–27]. Zhao et al.’s study harnessed the large sample size of the UKB cohort (n = 19,629) with the MRI data provided for each individual. They downloaded and processed MRI data to generate 101 different imaging traits including regional and total brain volumes (TBV) [28, 29]. They performed GWAS on the 101 imaging traits using 8,944,375 genetic variants. From this study, we used GWAS summary data on TBV—which is one of the 101 traits. GWAS summary data from this study may be found at: https://github.com/BIG-S2/GWAS.

We also performed our analyses using the GWAS summary data generated by Hibar et al. [13] from the ENIGMA consortium. This study was a volumetric meta-analytic GWAS which aimed to identify SNPs associated with seven subcortical brain structures and intracranial volume (ICV). The volume measures investigated in this study were obtained from structural MRI data (sample size n = 30,717), and then meta-analytic GWAS was performed on these volumetric phenotypes. From these results, we used only the GWAS summary data for ICV since it is a relevant brain volume trait that is similar to TBV mentioned above.

Summary statistics from both UKB and ENIGMA studies provide the required components for subsequent analyses with S-PrediXcan. These include SNP IDs, effect/non-effect alleles, standardized regression coefficients (BETA) and the associated *p*-values.

### Tissue-specific transcriptome analysis by using S-PrediXcan

S-PrediXcan is a method that estimates the mediating effects of gene expression levels on phenotypes using only GWAS summary data [18]. We applied S-PrediXcan to thirteen GTEx brain tissues [21] and two brain volume phenotypes (TBV and ICV). Input materials for our S-PrediXcan analysis included the TBV GWAS summary statistics from the UKB cohort (n = 19,629) [2] and the ICV GWAS summary statistics from the ENIGMA2 cohort (n = 30,717). All the genetic variants from the two GWAS summary statistics data were used in this study [13]. Another required input was the trained PrediXcan models [17] using elastic-net from the GTEx transcriptomes of thirteen brain tissues (GTEx version 8) [18], where each tissue-specific model predicts gene expression level in the corresponding brain tissue using relevant SNPs. In this work, we examined the trained PrediXcan models using the elastic-net method, while an interesting future direction would be to include also the MASHR-based PrediXcan models and perform a more comprehensive comparative study. The PrediXcan models and SNP covariances were downloaded from http://predictdb.org/. All the inputs were integrated through following the instructions in the “S-PrediXcan Input data” section available at https://github.com/hakyimlab/MetaXcan. Briefly, the MetaXcan repository was cloned to the local computer, and then the High-Level S-PrediXcan Script was run with the specified paths directed toward the corresponding input files. During the analyses, each of the thirteen brain tissue-specific prediction models was applied to predict genes that are associated with our interested GWAS traits (ICV and TBV).

S-PrediXcan was performed to integrate GTEx PrediXcan models with GWAS summary statistics data of our interested traits: (1) TBV from UKB and (2) ICV from ENIGMA2; and the goal was to identify tissue-specific transcriptomic variations commonly associated with TBV and ICV. The first S-PrediXcan analysis was done with the GWAS summary data from UKB cohort to identify genes that are highly associated with TBV. After that, the second S-PrediXcan analysis was performed with the GWAS summary data from the ENIGMA2 cohort, where our goal was to determine which TBV-associated genes are also significantly associated with ICV. The results were reported using a false discovery rate (FDR) threshold < 0.05 [30]. The schematic design of this work is shown in Fig. 1.

### Comparison with previous studies

The discovered genes were manually searched in the reports of previous relevant studies. The purpose of these searches was to determine which genes were previously reported to be associated with brain volumes and structures as well as associated with different cognitive traits and brain disorders. Another purpose was to confirm the validity of our analyses and also to point out which of the genes we discovered had not been reported in previous studies. One of the studies that we compared with was the gene-based association analysis of the original UKB GWAS where they reported 157 genes that were highly associated with different brain regional measures. We also manually checked our discovered genes in the NHGRI-EBI GWAS catalog (https://www.ebi.ac.uk/gwas/)-a database that contains prior GWAS findings. We wanted to check whether our discovered genes are correlated with any of the following cognitive deficits and mental-health related traits: neurodegenerative diseases, neuropsychiatric disorders, neuroticism, intellectual performance, reaction time, cognitive function, educational attainment and mathematical ability.

### Molecular and biological pathway investigations of the reported genes

To better understand the molecular mechanisms and biological pathways associated with our discovered genes, additional analyses were performed. The protein interaction network was obtained using STRING (https://string-db.org/). Pathway enrichment analysis was conducted using https://maayanlab.cloud/Enrichr/ to identify pathways that were enriched in our gene findings. We examined the pathways available in two different tracks (*molecular function* and *biological pathway*) of the Gene Ontology (GO) database.

## Supplementary Information

**Additional file 1**: This file contains the following supplementary tables. Supplemental Table S1a. Gene findings from the UKB analysis, where TBV is the trait of interest. Shown in the table are the p-values. Supplemental Table S1b. Gene findings from the ENIGMA2 analysis, where ICV is the trait of interest. Of note, this is a targeted analysis which only examines the gene findings from the previous UKB analysis. In other words, we were looking for which TBV-associated genes were also significantly associated with ICV. Shown in the table are the p-values. Supplemental Table S2. Among 10 genes discovered in our study, 9 of them (except FAM215B) are significantly associated with TBV in the gene-based association analysis of the original UKB GWAS (Zhao et al.). Supplemental Table S3. Results of enrichment analysis of 10 discovered genes on Gene Ontology Biological Processes. Supplemental Table S4. Results of enrichment analysis of 10 discovered genes on Gene Ontology Molecular Functions.

## Data Availability

GWAS summary data for the UKB study can be found at: https://github.com/BIG-S2/GWAS. GWAS summary data for the ENIGMA study can be found at: http://enigma.ini.usc.edu/research/download-enigma-gwas-results/. The PrediXcan models and SNP covariances can be found at: http://predictdb.org/.

## Abbreviations

GWAS: Genome-wide association study
SNP: Single nucleotide polymorphism
UKB: UK Biobank
ENIGMA: Enhancing Neuroimaging Genetics through Meta Analysis
TBV: Total brain volume
ICV: Intracranial volume
ROI: Region of interest
MRI: Magnetic resonance imaging
GTEx: The genotype-tissue expression project
FDR: False discovery rate
STRING: Search tool for recurring instances of neighbouring genes
GO: Gene ontology
